# Structural characterisation of human galectin-4 *N*-terminal carbohydrate recognition domain in complex with glycerol, lactose, 3′-sulfo-lactose, and 2′-fucosyllactose

**DOI:** 10.1038/srep20289

**Published:** 2016-02-01

**Authors:** Khuchtumur Bum-Erdene, Hakon Leffler, Ulf J. Nilsson, Helen Blanchard

**Affiliations:** 1 Institute for Glycomics, Griffith University, Gold Coast Campus, Queensland 4222, Australia; 2 Section MIG, Department of Laboratory Medicine, Lund University, BMC-C1228b, Klinikgatan 28, SE-22184 Lund, Sweden; 3 Centre for Analysis and Synthesis, Department of Chemistry, Lund University, PO Box 124, SE-22100 Lund, Sweden

## Abstract

Galectin-4 is a tandem-repeat galectin with two distinct carbohydrate recognition domains (CRD). Galectin-4 is expressed mainly in the alimentary tract and is proposed to function as a lipid raft and adherens junction stabilizer by its glycan cross-linking capacity. Galectin-4 plays divergent roles in cancer and inflammatory conditions, either promoting or inhibiting each disease progression, depending on the specific pathological condition. The study of galectin-4’s ligand-binding profile may help decipher its roles under specific conditions. Here we present the X-ray structures of human galectin-4 *N*-terminal CRD (galectin-4N) bound to different saccharide ligands. Galectin-4’s overall fold and its core interactions to lactose are similar to other galectin CRDs. Galectin-4N recognises the sulfate cap of 3′-sulfated glycans by a weak interaction through Arg45 and two water-mediated hydrogen bonds via Trp84 and Asn49. When galectin-4N interacts with the H-antigen mimic, 2′-fucosyllactose, an interaction is formed between the ring oxygen of fucose and Arg45. The extended binding site of galectin-4N may not be well suited to the A/B-antigen determinants, α-GalNAc/α-Gal, specifically due to clashes with residue Phe47. Overall, galectin-4N favours sulfated glycans whilst galectin-4C prefers blood group determinants. However, the two CRDs of galectin-4 can, to a less extent, recognise each other’s ligands.

Galectins are a family of soluble β-galactoside-specific lectins with high sequence similarity within their carbohydrate binding sites. The 15 known mammalian galectins can be classified into three distinct types according to the organisation of their carbohydrate recognition domains (CRDs): proto, chimera and tandem-repeat. Proto type galectins form homo-dimers through their single soluble CRD that comprises approximately 130 amino acids. The only member of the chimera type, galectin-3, has an *N*-terminal short peptide motif, a flexible collagen-like Gly/Pro/Tyr-rich domain, which induces self-oligomerization^1^, and a CRD at its *C*-terminus. The tandem-repeat galectins, of which galectin-4 is a member, contain two distinct CRDs at their *N*- and *C*-termini, connected by a variable-length linker region that is susceptible to proteolytic degradation^2^. The two CRDs of a tandem-repeat galectin are sufficiently different from one another that they can exhibit distinct binding preferences. Interest in galectins increased substantially since their discovery^3^ due to the variety of roles that they play in a number of disease states, including cancer^4^,^5^,^6^, heart disease^7^, inflammation^8^,^9^ and infection^10^.

Galectin-4 is found mostly in the gastrointestinal tract^4^ and its roles in the stabilisation of lipid rafts^11^,^12^,^13^,^14^,^15^ as well as the delivery and recruitment of glycoproteins to the lipid rafts^16^,^17^,^18^,^19^ at the apical brush border membrane of enterocytes have been well documented. The protein is able to cross-link combinations of sulfated glycosphingolipids, sulfated cholesterol, *N*- and *O*-glycosylated proteins and blood group antigens by its two domains to stabilise the lipid raft and its contents^16^,^18^,^20^,^21^,^22^. Galectin-4 has also been found to localise at the adherens junction of porcine oral epithelium, possibly acting as a stabiliser through its cross-linking capacity^23^. In addition to the gastrointestinal tract, galectin-4 is found in neuronal cells, acting as a regulator of myelination through the inhibition of oligodendrocyte maturation, which is possibly mediated through interactions with sulfatides as well as glycosylated axonal membrane L1^24^,^25^,^26^. In congruence with binding specificity studies indicating that galectin-4 does not recognise sialic acid^16^,^20^,^21^,^27^, galectin-4 does not interact with the GM1 gangliosides on human neuroblastoma cells and does not influence the proliferation and function of these cells^28^.

The involvement of galectin-4 in the inflammatory condition colitis is not well understood^29^. Earlier studies reported that galectin-4 exacerbates the condition and delays recovery from acute colitis^30^. However, a later study reported that galectin-4 had the capacity to reduce mucosal inflammation and induce T-cell apoptosis in a colitis model^31^. The opposing results could be reconciled by the finding that galectin-4 induced different effects on different model systems^32^. Galectin-4 expression is also correlated with inverted papillomas, along with galectin-3, -7 and -9^33^. The role that galectin-4 plays in malignant-tumor progression and metastasis is dependent on the specific type of cancer. Galectin-4 expression in liver and lung cancer leads to increased metastasis and cancer progression^34^,^35^. In contrast, its expression in colon and pancreatic cancers leads to a decrease in metastasis and cancer progression^36^,^37^,^38^,^39^,^40^; whilst expression in tongue cancer did not correlate with the disease progression^41^. Although further investigations of lesser-studied galectins, such as galectin-4, are ongoing, the therapeutic potential of galectin-1 and galectin-3 inhibitors have been well demonstrated^42^,^43^,^44^,^45^. Due to the complexity of the roles that galectins play in different stages of cancer progression, galectin-targeted treatments should be sufficiently specific to avoid neutralising the positive effects of those galectins, such as galectin-4, that have the ability to reduce cancer metastasis and progression.

The oligosaccharide-specificities of galectins may explain some of the observed differences in function among them. For example, galectin-3 and galectin-4 were localized at distinct parts of the T84 colon cancer cell membrane^46^. This recognition of different cell surface receptors indicates that the differing saccharide specificities of galectin-3 and galectin-4 might induce the pro- and anti-cancer roles of these galectins, respectively^46^. Characterisation of glycosylation patterns of specific cells and the binding specificities of galectins may lead to reasonable conclusions concerning the effect of galectin expression on the cell. Galectin-4 recognises saccharides with a sulfate cap at the non-reducing-end^20^,^27^,^47^. The ability of galectin-4 to recognise sulfatides allows its translocation and function in neuronal cells^25^,^26^. Lipid rafts in the brush border membrane of enterocytes are enriched in sulfoglycosphingolipids, through which galectin-4 is able to stabilise the complex^16^,^21^. Galectin-4 expression in the gastrointestinal tract along with its ability to recognise the SO_3_-Gal motif suggests that the lectin acts as a defense mechanism against infections by bacteria such as *H. pylori*, which specifically recognises 3′-sulfated galactose and has the ability to desulfate other sulfated saccharide motifs^16^,^21^,^27^,^48^,^49^. Both the *N*- and *C*-terminal CRDs of galectin-4 (galectin-4N and galectin-4C) can recognise sulfated saccharides^27^, though galectin-4N is more specific due to the presence of a non-conserved Arg45 residue that interacts with the sulfate moiety^22^. Structural characterization of galectin-4C bound to 3′-sulfated lactose (3′SuL) showed that a transient interaction between the sulfate group and the conserved Trp256 residue may help stabilise the complex, but no other direct interaction between the protein and the sulfate moiety was observed^50^.

The function of galectin-4 as a defence mechanism is further substantiated by the ability of galectin-4 to induce immunological response^30^,^51^ as well as the ability of galectin-4C to kill *E. coli* that express human blood group antigens^47^. Full-length human galectin-4 recognises blood group saccharides^27^,^47^ and galectin-4C recognises blood group A- and B- antigens more strongly than galectin-4N does, with the latter showing only marginal enhancement in affinity due to the presence of the non-reducing-end Gal/GalNAc^27^,^52^. Mutational analysis revealed that a non-conserved Phe47 residue of galectin-4N may be responsible for its inability to recognise A- and B-blood group antigens, specifically due to its bulky nature as the mutation Phe47Ala (making it then equivalent to that of galectin-4C) imbued high affinity towards A-tetrasaccharide while the Phe47Gln mutant failed to do so^52^. The equivalent of human galectin-4N Phe47 is found as His47 in mouse and rat galectin-4N. This change enhances the affinity of mouse galectin-4N towards B-antigen, possibly mediating interactions through the histidine whilst the larger *N*-acetyl group of the A-antigen may find that this amino acid is still too bulky and clash^53^. Similarly, rat galectin-4N gains only marginal affinity towards A- and B- linear trisaccharides compared to the lactose disaccharide^54^. The H-antigen with galactose 2′-fucosylation is recognised by both rat and mouse galectin-4N^53^,^54^, suggesting a binding motif where the H-antigen trisaccharide is recognised but the non-reducing-end α-linked Gal/GalNAc of B-/A-antigens are only tolerated, possibly imparting marginal enhancement in binding affinity. In contrast to the increased affinity of galectin-4C toward H-antigen, our structural analysis of galectin-4C with 2′-fucosyllactose showed that the fucose residue did not interact with galectin-4C, while molecular dynamics simulations suggested that the non-reducing-end α1-3-linked GalNAc/Gal residues of A- and B-antigens interact with galectin-4C, increasing binding affinity^50^.

The *N*- and *C*-terminal CRDs of human galectin-4 bear resemblance to the human galectin-8 *N*- and *C*-terminal CRDs, respectively, in their ability to recognise sulfated saccharides by the *N*-terminal CRDs^52^ and the ability to bind blood group saccharides by the *C*-terminal CRDs^47^. In fact, both galectin-4C and galectin-8C are necessary and sufficient to kill bacteria expressing blood group antigens^47^. One distinct difference between galectin-4N and galectin-8N is the inability of galectin-4N to recognise sialylated glycans, while galectin-8N specifically recognises sialic acid via its non-conserved Arg59 residue, which galectin-4N lacks^52^. Mutation of the bulky Phe47 residue of galectin-4N to Gln, as found in galectin-8N at the equivalent position slightly increased the affinity of galectin-4N toward 3′-sialyllactose, but the binding affinity was still 4-fold lower than that of lactose^52^. The intense work regarding the structural characterisation of galectin-8 has been integral to the understanding of its ligand recognition and biological function.

Unlike galectin-8, structural characterisation of galectin-4 has been limited, where the X-ray crystal structures of galectin-4C with its ligands have been only recently published^50^. Structural characterisation of galectin-4N has thus far been limited to two X-ray crystal structures of mouse galectin-4N (PDB ID: 3I8T^55^, 2DYC (Kato-Murayama *et al.*, unpublished)) and a crystallisation communication of His-tagged human galectin-4N without structure coordinates^56^. Here we report the first X-ray structures of human galectin-4N to shed light on its ability to recognise sulfated saccharides and its ability to recognise the fucose residue of H-antigen. The crystal structure exhibits different unit cell parameters and is crystallised under different conditions compared to those reported by Zimbardi *et al.* in their preliminary crystallisation report^56^. Our elucidation of the structure of human galectin-4N and ligands provides the basis for further ligand interaction studies and structure-based drug design as well as giving insight into the molecular basis of galectin-4 recognition of its ligands.

## Results and Discussion

### Overall structure of galectin-4N

To investigate the interaction between human galectin-4N and its natural saccharide ligands, we have determined the crystal structures of human galectin-4N in complex with glycerol (the cryoprotectant), lactose, lactose-3′-sulfate (3′SuL), and 2′-fucosyllactose (2′FL) at 1.70–2.00 Å resolution (Table 1). The crystals belong to space group P2_1,_ contain 4 monomers in the asymmetric unit and represents a different crystallographic system than for the mouse galectin-4N structures and also that described within the preliminary crystallisation report of the His-tagged human galectin-4N crystal^55^,^56^. The electron density defines amino acids 14–153 (protein construct being 1–154), the flexibility of the *N*-terminal residues and the *C*-terminal linker portion may explain the lack of electron density for these residues. The saccharide ligands studied herein are not significantly influenced by crystallographic contacts, though any influences on the binding site are described in detail in analysing each complex.

**Figure 1:**
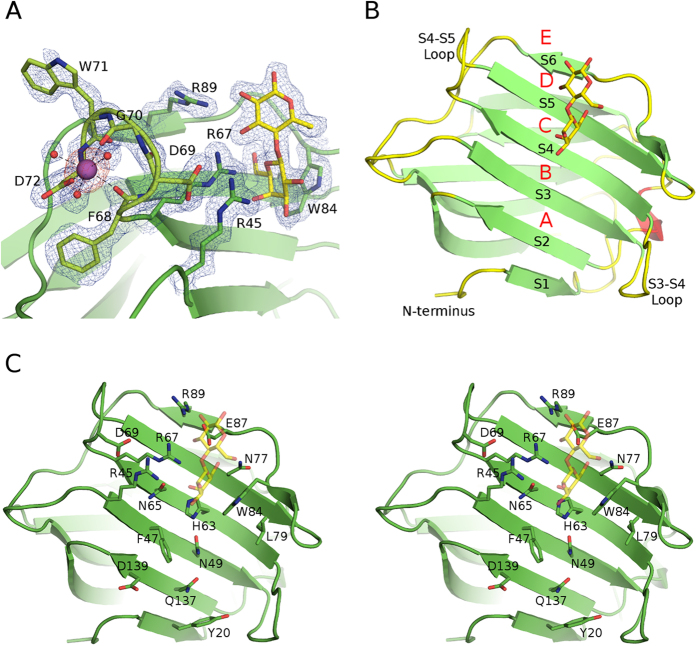
Structure of galectin-4N. (**A**) Simulated annealing omit map of the calcium-binding site (engaging the S4–S5 loop) of the galectin-4N CRD with bound lactose. Galectin-4N CRD displayed as green cartoon representation with calcium- and lactose-binding site residues depicted in green sticks. Calcium ion is shown as a magenta sphere, its coordinating waters (red spheres) and the bound lactose molecule (stick model with carbon atoms in yellow). The S4–S5 loop cartoon and carbons are highlighted in lime green. Dashed lines highlight calcium coordination with its surroundings. The 2|Fo| − |Fc|α_calc_ map is illustrated in blue mesh at a 1.0 σ contour level and the |Fo| − |Fc|α_calc_ map is illustrated in red mesh at a 3.0 σ contour level. The omit map was created by Phenix. (**B**) Galectin-4N CRD shown in cartoon representation (β-strands (green), α-helical turn (red), loops (yellow)) with bound lactose molecule in stick representation (yellow carbons, red oxygens). The β-strands on the concave side of the β-sandwich and certain loops are labelled in black. Binding sub-sites are labelled in red. (**C**) Stereo image of galectin-4N CRD with side-chains of residues implicated in recognition of oligosaccharides displayed in stick representation (carbon (green) oxygen (red), nitrogen (blue)). Bound lactose molecule in transparent stick representation (carbon (yellow), oxygen (red)) indicates the conserved carbohydrate-binding site.

Crystal packing of the monomers within the asymmetric unit is different from that of the cation-mediated tetramer observed in the mouse galectin-4N structure^55^. However, due to the presence of calcium acetate in the crystallisation condition of the galectin-4N-lactose complex, the four galectin-4N monomers each contained a bound Ca^2+^ ion, coordinated by the backbone carbonyls of Phe68 and Gly70, the carboxylate of Asp72 and 3 water molecules (Fig. 1A). The orientations of the residues involved in the coordination of the Ca^2+^ ion are conserved in the other galectin-4N complex structures and harbour a globular density indicative of an atom/ion with fewer electrons. As the protein preparation and the crystallisation procedure of crystals other than the galectin-4N-lactose complex did not use Ca^2+^-containing buffers and since the electron density at these positions is weaker than expected for a Ca^2+^ ion, the possible occupants of these positions were narrowed to be water molecules or potentially a Na^+^ ions in these structures. Unlike the galectin-4N-lactose complex structure, where the Ca^2+^ ions were coordinated by 3 water molecules in addition to the protein residues, the majority of these sites lacked the 6-coordination necessary for a Na^+^ ion. Due to the weak electron density and the lack of proper coordination, we have assigned water molecules at these positions for the galectin-4N-glycerol, galectin-4N-3′SuL and galectin-4N-2′FL complex structures, despite the high concentration of Na^+^ ions in the crystallisation condition. Further studies are necessary to establish whether the presence of the Ca^2+^ ion is a crystallographic artefact or it might have biological implications. Of relevance is that as a stabilizer of adherens junctions and lipid rafts, galectin-4 comes into contact with significant concentrations of calcium, whereby this ion also contributes to the stabilisation of adherens junctions via cadherins and calcium signalling is transduced through channels localized on lipid rafts^57^,^58^. It could be postulated that high concentration of calcium could induce stabilization of the S4–S5 loop of galectin-4N and increase its binding affinity towards its glycan binding partners, which could lead to further stabilization of lipid rafts and adherens junctions.

The overall fold of human galectin-4N is the conserved galectin β-sandwich fold comprising two β-sheets, F1–F5 and S1–S6, of which the concave side of the β-sandwich (S1–S6) contains the saccharide-binding site (Fig. 1B). As with other galectin CRDs, the amino acids forming the binding sub-sites C and D are conserved, while those that construct the A, B and E sub-sites are slightly different, granting galectin-4N its unique binding specificity (Fig. 1C). The human and mouse galectin-4N proteins exhibit high amino acid sequence identity (82.23% (residues 1–152) and comparison of their structures (PDB ID: 3I8T^55^, 2DYC (Kato-Muramaya *et al.* unpublished)) showed Cα RMSDs of 0.67 Å (chain A of galectin-4N-glycerol complex compared with 3I8T) and 0.75 Å (chain A of galectin-4N-glycerol complex compared with 2DYC). The most significant deviations were seen around the flexible S3–S4 loop region. Residues Arg89 (mouse Lys89), Leu79 (mouse Met79), Pro56 (mouse Asp56), Phe47 (mouse His47) and Tyr20 (mouse Lys20), which may have implications in saccharide recognition, are not conserved between the species.

The loop regions of galectins and the residues located within those loops are important for the binding specificity of each galectin. In particular, the uniquely long S4–S5 loop of galectin-1 protrudes into the saccharide-binding site and interacts with certain ligands, such as TDG^59^, but in contrast, the equivalent galectin-4N loop (shorter than that of galectin-1 and is a similar in size to that of galectin-3) does not directly interact with the ligands studied herein. Galectin-4N Asp69, located on the S4–S5 loop, is found in a position to interact with Arg45 (2.8–2.9 Å distance), keeping the guanidino moiety of the arginine in a stacking position to the conserved Arg67 (Fig. 1C). The Asp69 is semi-conserved among certain galectins as a negatively charged residue (galectin-7 Asp55, galectin-9N Glu67, galectin-9C Asp241) to interact with the equivalents of galectin-4N Arg45 (galectin-7 Arg31, galectin-9N Arg44, galectin-9C Arg221). This charge-charge interaction keeps the Arg residues stacked on top of each other and does not allow extended freedom to their sidechains. Galectin-3 and galectin-8N lack the negatively charged residue at the equivalent position of galectin-4N Asp69 (galectin-3 Asn164, galectin-8N Lys71) despite having an equivalent of galectin-4N Arg45 (galectin-3 Arg144, galectin-8N Arg45). This results in galectin-3 having a unique larger groove near the binding site, which has been the target of selective inhibition strategies^60^,^61^. The cationic Arg45 and Lys71 of galectin-8N do not form an attractive charge-charge interaction, which allows the Arg45 to position optimally for interactions with negatively charged sulfate and sialic acid groups^52^.

The S3–S4 loops of galectins are rather long and can be involved in binding with oligosaccharides (Fig. 1B). In galectin-9N recognition of poly-lacNAc, the loop interacted with the non-reducing-end of the linear polysaccharide^62^. In the galectin-8N recognition of sulfate and sialic acid groups, a non-conserved Arg59 stemming from this loop interacted with the negatively charged groups^52^; and in galectin-4C recognition of LNnT, the Lys226 from this loop interacted with the non-reducing-end galactose^50^. The S3–S4 loop region of galectin-4N does not contain any residues that could reach and interact with the particular ligands studied herein. However, the loop may be able to interact with larger oligosaccharides such as poly-lacNAc in the same manner as seen in galectin-9N^62^.

### Galectin-4N-glycerol complex

**Figure 2:**
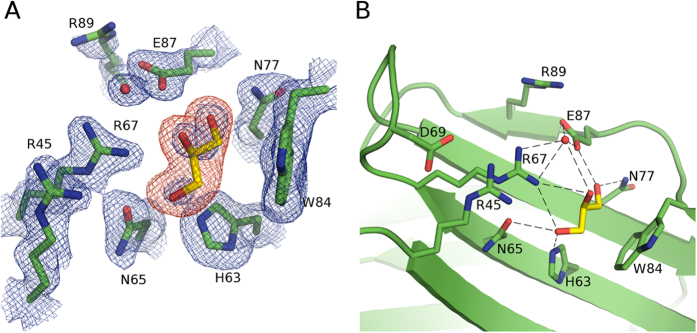
Crystal structure of galectin-4N-glycerol complex. (**A**) Simulated annealing omit map of galectin-4N binding site with bound glycerol. Galectin-4N binding site residues (green carbons) with bound glycerol (carbon (yellow)) depicted in stick representation. The 2|Fo| − |Fc|α_calc_ map is illustrated in blue mesh at a 1.0 σ contour level and the |Fo| − |Fc|α_calc_ map is illustrated in red mesh at a 3.0 σ contour level. The omit map was created by the “composite omit map” task of Phenix^89^. (**B**) Binding site interactions of galectin-4N and glycerol. Galectin-4N binding site depicted in green cartoon representation with binding site residues and bound glycerol (carbon (yellow)) represented as sticks. Hydrogen bonds are illustrated as dashed lines.

The cryo-protectant glycerol was able to soak into the binding site of the apo-crystal. The galectin-4N-glycerol complex was determined at 2.00 Å resolution. The binding sites of all 4 of the monomers displayed clear density for the bound glycerol molecule (Fig. 2A). The binding of the glycerol molecule is not influenced by crystallographic contacts in any of the 4 monomers.

The oxygen atoms of the glycerol molecule are found in positions that mimic galactose O4′, O5′ and O6′ and interact with the conserved residues His63, Asn65, Arg67, Asn77 and Glu87 (Fig. 2B). The carbon atoms of the glycerol molecule mimic the cyclic ring of galactose and make a van der Waals interaction with Trp84. In addition to the glycerol molecule, a water molecule is found in interaction distance of Arg67 and Glu87 residues and mimics the conserved interaction observed between lactose C3 hydroxyl and these two protein residues. This binding profile of glycerol mimicking galactose and a water molecule occupying the binding site of glucose C3 hydroxyl is also seen in galectin-3 (PDB ID: 3ZSK^63^) and galectin-8C (PDB ID: 4GXL^64^).

In our human galectin-4N-glycerol complex structure, the binding site of galectin-4N is pre-organised to interact with saccharides. This could be due to the ability of glycerol to mimic saccharides as a number of NMR studies suggest that the side-chains of galectins that are involved in saccharide binding have much greater conformational freedom (PDB ID: 2KM2^65^, 2YRO (Tomizawa *et al.* unpublished), 1×50 (Tomizawa *et al.* unpublished)). The mouse apo-galectin-4N structure (PDB ID: 2DYC, Kato-Muramaya *et al.* unpublished) also has a pre-organized binding site, but further scrutiny suggests that the 3 water molecules modelled in its binding site may be a glycerol molecule, as the crystallisation condition contains a high percentage of glycerol. Importantly, in the human galectin-4N-glycerol structure, the interaction between Asp69 and Arg45 still forms in the absence of a natural saccharide ligand (Fig. 2B). The equivalent interaction is not kept in either the mouse apo-galectin-4N structure (PDB ID: 2DYC (Kato-Muramaya *et al.* unpublished)) or the mouse galectin-4N-Lac complex (PDB ID: 3I8T^55^). As the sequence of the S4–S5 loop and the residues that coordinate the loop are identical between the human and mouse galectin-4N, the difference may be due to crystallographic contacts. The Ca^2+^ coordinating sites of both the apo- and holo- mouse galectin-4N structures harbour a crystallographic contact to Lys97 and the S4–S5 loop may be pulled slightly further away. The S4–S5 loop of the human galectin-4N structures are relatively less influenced by crystal contacts and as such reveal the important Arg45-Asp69 interaction as well as its ability to coordinate a cation. The Arg45-Asp69 interaction may have an effect on the ability of Arg45 to move away from this position and interact with the sulfate group of sulfated saccharides in a matter that has been predicted by mutational analysis^22^.

### Galectin-4N-lactose complex

**Figure 3:**
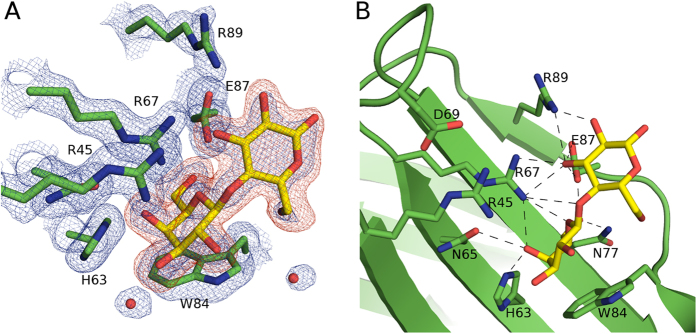
Crystal structure of galectin-4N-lactose complex. (**A**) Simulated annealing omit map of galectin-4N binding site with bound lactose. Galectin-4N binding site residues (green carbons) with the bound lactose (yellow carbon) depicted in stick representation. The 2|Fo| – |Fc|α_calc_ map is illustrated in blue mesh at a 1.0 σ contour level and the |Fo| – |Fc|α_calc_ map is illustrated in red mesh at a 3.0 σ contour level. The omit map was created by the “composite omit map” task of Phenix^89^. (**B**) Binding site interactions of galectin-4N and lactose. Galectin-4N binding site depicted in green cartoon representation with binding site residues (carbon (green)) and bound lactose (carbon (yellow)) represented as sticks. Hydrogen bonds are illustrated as dashes.

The galectin-4N-lactose complex structure was determined at 1.90 Å resolution from crystals grown in a PEG-based condition that is different to the other crystals reported here (which were crystallised in high concentration of sodium formate). However, the space group and unit cell dimensions are comparable (Table 1). The binding sites of all 4 monomers within the asymmetric unit contained unambiguous density for the lactose molecule (Fig. 3A). In the binding sites of monomers A and C, the C2′ hydroxyl of the galactose residue of lactose is in crystal contact with the backbone carbonyl of Gly82 from another monomer. In monomers B, C, and D the C1 and C2 hydroxyls of the glucose residue of lactose are in contact with the Asp55 of another monomer, while the A monomer is free of any other crystal contact. We consider that these crystal contacts do not have influence on the binding mode of lactose, especially in the case of the lactose in monomer A, because in monomers B and D, which do not interact with the backbone carbonyl of Gly82, the location of this interaction is replaced by a water molecule that mediates a hydrogen-bond with Arg45. The location of this water molecule is also replaced by the ring oxygen of fucose in the galectin-4N-2′FL complex.

The binding interactions observed between galectin-4N and lactose are the same as the conserved interactions observed in previously reported galectin-lactose complex structures (Fig. 3B). The C4′ hydroxyl of the galactose saccharide interacts with His63, Asn65 and Arg67. The C6′ hydroxyl of the galactose residue interacts with Asn77 and Glu87. The ring oxygen of galactose is in interaction distance of Arg67. The C3 hydroxyl of the glucose residue is in interaction distance of Arg67, Glu87 and Arg89. Additionally, the C2 hydroxyl of glucose is in hydrogen bonding distance of Arg89. These interactions form the core of the conserved galectin-saccharide interaction, with the exception of Arg89, which is a semi-conserved residue among human galectins. The Arg89 confers additional interactions with the C2 and C3 hydroxyls of lactose as seen here, and the galectin-3 equivalent (Arg186) increases the affinity of *N*-acetyllactosamine towards galectin-3^66^. Within the asymmetric unit, Arg89 interacts with lactose only in monomer A, but instead forms crystallographic salt bridges with Asp55 of other monomers in monomers B, C and D. As we observed in the interactions between the galectin-3 Arg186 (equivalent to galectin-4N Arg89) and other saccharide ligands^67^, the galectin-4N Arg89 interaction with lactose and similar ligands may be transient.

Although the core binding site of galectins (sub-sites C and D) are largely conserved among galectin CRDs, interacting in a similar manner with basic ligands like lactose, differences in binding affinity have been reported^68^. In the case of galectin-4N, the affinity for lactose (1300 μM; Table 2) is slightly higher than that of the galectin-4C (1900 μM), which we reported previously using the same assay^50^. The presence of galectin-4N Arg89 which can form transient interactions with the lactose C2 and C3 hydroxyls could contribute to the slightly higher affinity, as the galectin-4C equivalent at this position is Lys261, which does not form similar interactions^50^.

### Galectin-4N-lactose-3′-sulfate complex (3′SuL)

**Figure 4:**
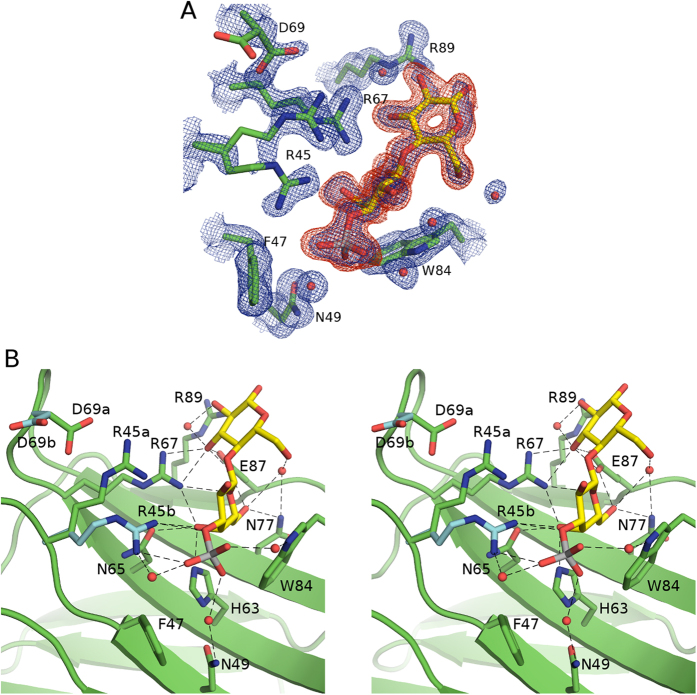
Crystal structure of galectin-4N-3′SuL complex. (**A**) Simulated annealing omit map of galectin-4N binding site with bound 3′SuL. Galectin-4N binding site residues (carbon (green)) with the bound 3′SuL (carbon (yellow), sulphur (silver)) depicted in stick representation. The 2|Fo| – |Fc|α_calc_ map is illustrated in blue mesh at a 1.0 σ contour level and the |Fo| – |Fc|α_calc_ map is illustrated in red mesh at a 3.0 σ contour level. The omit map was created by the “composite omit map” task of Phenix^89^. (**B**) Binding site interactions of galectin-4N and 3′SuL. Stereo image of galectin-4N binding site depicted in green cartoon representation with binding site residues (carbon (green); alternative conformations of Arg45 and Asp69 are illustrated with carbon (cyan)) and bound 3′SuL (carbon (yellow)) represented as sticks. Hydrogen bonds are illustrated as dashes.

The galectin-4N-3′SuL complex structure was determined at 1.70 Å resolution from an apo-galectin-4N crystal soaked in 20 mM 3′SuL (Table 1). Clear electron densities were observed for the bound ligand in all 4 monomers (Fig. 4A). The 3′SuL ligand in the binding site of monomer A makes crystallographic contacts with the backbone carbonyl of Gly82 of monomer B via galactose O2′ and side-chain of Asp55 of monomer C via glucose O6. The ligand in monomer B makes crystallographic contacts with the side-chain of Asp55 of monomer D via glucose O1/O2 and side-chain of Gln54 of monomer C via glucose O6. The ligand in monomer C makes crystallographic contact with the backbone carbonyl of Gly82 of monomer D via galactose O2′. The ligand in monomer D makes crystallographic contact with the side-chain of Asp55 of monomer A via glucose O1/O2. As with the galectin-4N-lactose complex, we do not think these crystal contacts induced changes within the binding interaction, and structural alignment of the monomers show identical binding interactions and ligand conformations.

The interactions between the galectin-4N binding site and the lactose moiety of 3′SuL are comparable to those found in the galectin-4N-lactose complex (Fig. 4B). The sulfate moiety interacts with Trp84 and Asn49 via two water-mediated hydrogen bonds that are preserved in all 4 of the monomers. In a dynamic binding environment, the sulfate residue may also be able to directly interact with Trp84 ring nitrogen by rotating about the sulfate–galactose C3′ bond, as seen in the complex of galectin-4C–3′SuL^50^. Interestingly, only the binding site electron density of monomer A clearly supported an alternative conformation for Arg45 that allows direct interaction with the sulfate group (Fig. 4B). The difference electron density around the B-subsite (sulfate interaction site) of the other three monomers showed slight hints toward secondary alternative conformations of Arg45 with very low occupancies, but the experimental evidence was not as clear as that of monomer A. The conformation of Arg45 of monomer A is not due to crystallographic contacts, because the conformation of Arg45 of monomer A, which does not interact with the sulfate group, is in interaction distance of the backbone carbonyl of monomer B Gly82 residue (3.2 Å). This interaction would encourage restraint of Arg45 in the non-sulfate-interacting conformation thus discouraging the interaction between Arg45 and the sulfate group. Despite this crystallographic contact, the alternative conformation of Arg45 that interacts with the sulfate group is clearly supported by the electron density. Therefore it is our interpretation that the residue Arg45 is in equilibrium between hydrogen bonding with the sulfate group of 3′SuL and the residue Asp69. The crystal structure shows that the Arg45 residue is capable of interacting with the sulfate residue and also depicts the orientation of the residue required for the interaction (Fig. 4B).

Unlike galectin-8N, which binds to SO_3_^−^-Lac-pNP with 34-fold higher affinity than Lac-pNP^69^, the reported affinity of galectin-4N toward SO_3_^−^-Lac-pNP is only 5.4-fold higher than Lac-pNP^52^, while others have reported slightly higher ratio of 10–20 fold^27^. Our affinity data also shows 2.6-fold higher affinity for 3′SuL compared to lactose (Table 2), supporting the former data. Notably, the affinity of galectin-4N toward 3′SuL (510 μM) is 2.7-fold higher than that of galectin-4C (1400 μM)^50^, which also agrees with previously published data indicating that galectin-4N recognises sulfated saccharides with higher affinity and specificity than galectin-4C^22^,^27^. The 34-fold higher affinity of galectin-8N towards the sulfate group is likely due to the number of specific interactions afforded by the protein through residues Arg45, Arg59, Gln47 and Trp86 (equivalent to galectin-4N Arg45, no equivalent, Phe47 and Trp84 respectively)^52^ while galectin-4N recognition of the sulfate group only employs direct interaction via Arg45 and two water-mediated hydrogen-bonds to Trp84 and Asn49. Additionally, the Arg45 residue of galectin-8N (galectin-4N Arg45) is more readily available for interaction with the sulfate group due to the presence of a non-negatively charged residue Lys71 (galectin-4N Asp69) which does not trap the Arg45 residue in a non-interaction conformation as observed in galectin-4N through the Arg45-Asp69 interaction (Fig. 4B).

### Galectin-4N-2′-fucosyllactose complex (2′FL)

**Figure 5:**
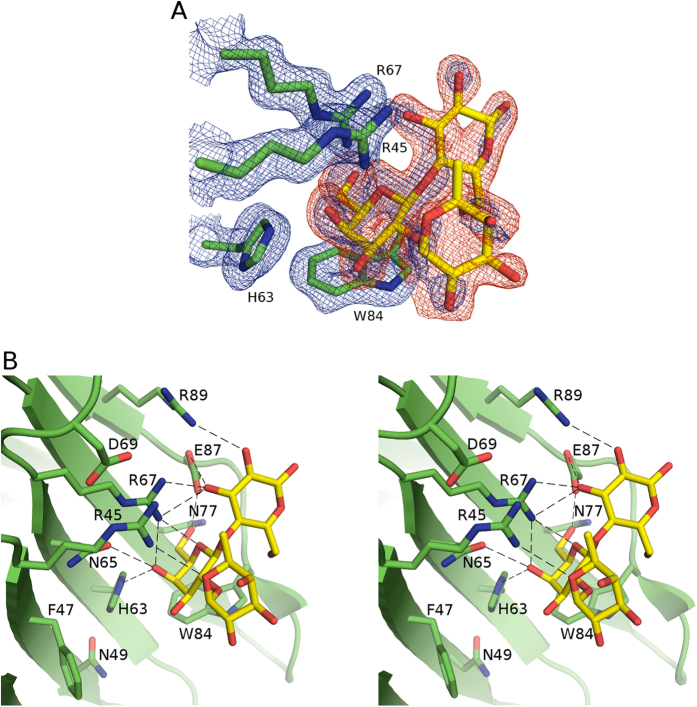
Crystal structure of galectin-4N-2′FL complex. (**A**) Simulated annealing omit map of galectin-4N binding site with bound 2′FL. Galectin-4N binding site residues (carbon (green)) with the bound 2′FL (carbon (yellow)) depicted in stick representation. The 2|Fo| – |Fc|α_calc_ map is illustrated in blue mesh at a 1.0 σ contour level and the |Fo| – |Fc|α_calc_ map is illustrated in red mesh at a 3.0 σ contour level. The omit map was created by the “composite omit map” task of Phenix^89^. (**B**) Binding site interactions of galectin-4N and 2′FL. Stereo image of galectin-4N binding site depicted in green cartoon representation with binding site residues (carbon (green)) and bound 2′FL (carbon (yellow)) represented as sticks. Possible hydrogen bonds are illustrated as dashes.

The galectin-4N-2′FL complex structure was determined at a resolution of 1.85 Å from a ligand-soaked apo crystal (Table 1). The ligand was able to enter into the relatively open binding sites of monomers B and D, where clear electron density was observed for the ligands (Fig. 5A). In contrast, the binding sites of monomers A and C were more occluded by crystal contacts and the cryoprotectant glycerol was found within the binding sites. The 2′FL in monomer B is in crystallographic contact with Asp55 of monomer D via glucose O1/O2 and Gln54 of monomer C via glucose O6. The ligand in monomer D is in crystallographic contact with Asp55 of monomer A via glucose O1/O2 and no other crystal contacts were observed, thus monomer D and bound 2′FL are used for analysis.

As with the galectin-4N-3′SuL complex, the interactions between galectin-4N and the lactose portion of 2′FL are comparable to those observed in the galectin-4N-lactose complex (Fig. 5B). Unlike previous galectin complex structures with oligosaccharides containing the 2′FL motif (PDB ID: 4YM1^50^, 1ULF^70^, 3WG3^71^) the fucose makes a direct interaction with galectin-4N Arg45 residue by its ring oxygen (Fig. 5B). The additional interaction is consistent with our affinity data, which shows 3.4-fold higher affinity for the galectin-4N-2′FL interaction compared to the galectin-4N-lactose interaction (Table 2). Furthermore, galectin-4N affinity toward 2′FL (380 μM) is 1.5-fold higher than that of galectin-4C (580 μM^50^).

Unlike the recognition of sulfated saccharides, the ability of human galectin-4N to recognise blood group saccharides has been suspect to controversy. Solid-phase competition assay suggested that galectin-4N does not bind any of the blood group antigens^27^, but surface plasmon resonance studies have shown 2-20-fold higher affinity for the galectin-4N-A-tetrasaccharide interaction compared to galectin-4N-lactose interaction^21^,^52^. Our affinity data supports the results from the latter study (Table 2), where the affinity of A-tetrasaccharide toward galectin-4N (164 μM) is 7.9-fold higher than that of lactose (1300 μM). Furthermore, as with the surface plasmon resonance study, which reported 1.6-fold higher affinity for galectin-4C-A-tetrasaccharide compared to galectin-4N-A-tetrasaccharide^21^, our data also shows 2.2-fold higher affinity for human galectin-4C toward A-tetrasaccharide (75 μM^50^) compared to human galectin-4N. Further scrutiny of the affinity data reveals that galectin-4N affinity toward A-tetrasaccharide (164 μM) is only 2.3-fold higher than toward 2′FL (380 μM), whilst galectin-4C affinity toward A-tetrasaccharide (75 μM) is 7.7-fold higher than toward 2′FL (580 μM), which indicates that the non-reducing-end GalNAc interacts well with the galectin-4C protein surface as described previously^50^, while its interaction with galectin-4N surface maybe less optimal. The B sub-site of galectin-4N, in which the GalNAc of A-tetrasaccharide would interact, is very different to the B sub-site of galectin-4C, lacking the galectin-4C Ser220 residue (galectin-4N Arg45) that interacts with the *N*-acetyl moiety of GalNAc, as well as having a bulky Phe47 residue. If the non-reducing end GalNAc of A-tetrasaccharide could orient in the binding site of galectin-4N in a similar to that of galectin-4C, the additional 2.3-fold affinity may be imbued by the interaction between the GalNAc C6 hydroxyl and the Trp84.

The rat and mouse galectin-4N both recognise at least the H-antigen (2′FL) with higher affinity than lactose and the mouse galectin-4N recognises B-antigen strongly^53^,^54^. The rat galectin-4N also recognises both A- and B- linear trisaccharide groups with slightly higher affinity than lactose^54^. The higher affinity toward these saccharides is consistent with our affinity and crystallographic data regarding the human galectin-4N. The ability of rat and mouse galectin-4N to recognise the A- and B-antigens may be further enhanced by the presence of a His residue instead of the human galectin-4N Phe47 residue. The histidine can interact directly with the non-reducing-end galactose of B-antigen; and, at least in the case of B-antigen, the non-reducing-end antigenic galactose residue may not clash with this His residue of rat and mouse galectin-4N, resulting in a significantly higher affinity^53^. Mutation Phe47Ala increased the affinity of galectin-4N toward A-tetrasaccharide by 4.4-fold, while Phe47Gln mutation decreased the affinity 3.7-fold^52^. This suggests that the size of the side-chain might be sterically hindering GalNAc from binding, and the hydrophobicity of Phe47 may not be the main cause. In fact, it can be hypothesized that the 3.7-fold loss of affinity may be due to a loss of a stacking interaction between Phe47 and the N-acetyl group of GalNAc. The galectin-4C equivalent of galectin-4N Phe47 is Ala222, which allows the non-reducing-end GalNAc or Gal of A- and B-antigens to situate within the binding site of galectin-4C without steric hindrance^50^.

Overall, the structural and binding affinity analysis of human galectin-4N in complex with 2′FL reveals that galectin-4N recognises H-antigen with higher affinity than galectin-4C and recognises A- and B-antigens to a lesser extent than galectin-4C. The limitation in recognising A- and B- antigens may be due to the B-subsite of galectin-4N being less than ideal for accommodating the non-reducing-end Gal/GalNAc of B- and A-antigens. However, the ability of each galectin-4 CRD to be able to recognise glycan containing blood group determinants may be an adaptation to its environment within the intestinal tract as glycopeptides and glycolipids of intestinal epithelial cells have higher than average blood group determinant content^72^,^73^.

## Concluding remarks

Here we report the first X-ray crystal structures of human galectin-4N with bound glycerol, lactose, 3′SuL and 2′FL. The overall structure of human galectin-4N CRD is similar to those observed previously for other galectins, and as with these prior structures galectin-4N recognises lactose via the conserved residues within the C, D and E sub-sites (Fig. 3B). Galectin-4N recognises the sulfate group of 3′SuL by water-mediated hydrogen-bonding interactions with Asn49 and Trp84 (Fig. 4B). The Arg45 residue is found to weakly interact with the sulfate group, possibly enhancing the binding affinity further. The galectin-4N Arg45 interaction with the sulfate of 3′SuL may be weakened by the semi-conserved Asp69 residue that traps Arg45 in a charge-charge interaction (Fig. 4B), creating an equilibrium between Arg45-sulfate and Arg45-Asp69 interactions. Furthermore, the interaction between Arg45 and Asp69 of galectin-4N may be responsible for the inability of taloside-based inhibitors to bind galectin-4N as the Arg45/Arg67 stacked conformation blocks the intended binding groove for taloside C2 modifications^60^,^61^. Galectin-4N recognises the fucose group of 2′FL by its ring oxygen through an interaction with Arg45 (Fig. 5B). The bulky Phe47 residue in the B sub-site of galectin-4N may be responsible for its lower affinity toward A- and B-antigens, possibly interfering with the linear portion of these antigens.

Parallels can be drawn between galectin-4 and galectin-8, attributing each domain to a specific set of oligosaccharides such as sulfated saccharides and blood group antigens, but galectin-8 is a specialized member of the tandem-repeat galectins due to its high-affinity for sulfated and sialylated saccharides via its *N*-terminal CRD and specific recognition of blood group antigens via its *C*-terminal CRD^69^, where one CRD cannot recognise the ligand of the other. On the contrary, galectin-4 *N*- and *C*-terminal CRDs recognise each other′s respective binding partners with slightly lower affinity^20^,^21^,^50^, which allows each domain to recognise a slightly more diverse set of oligosaccharides. The ability of galectin-4 CRDs to recognise and bind a larger repertoire of ligands with slightly lower affinity than the individual CRDs of galectin-8 may be a unique distinction, through which galectin-4 may function. A conjecture may be that the cellular environment, with increasing or decreasing concentrations of glycosphingolipids or glycoproteins, may alter the capabilities of galectin-4 as a cross-linker. This may in part explain the negative effects of galectin-4 over-expression in cancers outside of the intestinal tract^34^,^35^, as the protein may induce unintended effects via glycan interactions that is not part of its natural repertoire.

## Materials and Methods

### Materials

β-lactose and lactose-3′-sulfate were purchased from Sigma (St. Louis, MO, USA) and 2′-fucosyllactose was purchased from Dextra Laboratories (Reading, UK).

### Purification, crystallisation and soaking of galectin-4N

Expression and purification of galectin-4N was performed using a lactosyl-Sepharose affinity chromatography as established for galectin purification^74^. Galectin-4N-lactose complex crystals were grown by the hanging-drop vapor-diffusion method with 500 μl reservoir solution (0.2 M calcium acetate and 20% w/v PEG 3350) and a 4 μl drop containing 2 μl protein solution (7 mg/ml in 150 mM NaCl, 50 mM HEPES pH 7.2 and 50 mM β-lactose) and 2 μl reservoir solution at 20 °C. The crystals grew in 20–30 days to an average size of 0.2 mm × 0.2 mm × 0.1 mm. Apo-galectin-4N crystals were obtained by the hanging-drop vapor-diffusion method with 500 μl reservoir solution (3.5 M sodium formate, 0.1 M Tris pH 8.0) and a 4 μl drop containing 2 μl protein solution (5 mg/ml in PBS pH 7.4) and 2 μl reservoir solution at 20 °C. The crystals grew to an average size of 0.04 mm × 0.04 mm × 0.02 mm in 30–40 days. Galectin-4N-2′FL and galectin-4N-3′SuL complexes were generated by soaking the apo-galectin-4N crystals in soaking solutions (4.0 M sodium formate, 0.1 M Tris pH 8.0) supplemented with 20 mM 2′FL or 3′SuL for 60–70 min. The cryo-protectant glycerol is found in the binding site of the apo-galectin-4N structure, creating the galectin-4N-glycerol complex.

### X-ray data collection and structure determination

All X-ray data collection experiments were conducted at the Australian Synchrotron MX1 and MX2 beamlines. Galectin-4N crystals were dipped in cryo-protectant solutions (30% w/v PEG 3350, 0.2 M CaCl_2_, 7% v/v glycerol for galectin-4N-Lactose complex and 4.0 M sodium formate, 0.1 M Tris pH 8.0, 10% v/v glycerol for others) before being flash-cooled in liquid nitrogen. The X-ray diffraction data were indexed and integrated using XDS^75^,^76^ or iMOSFLM^77^ and scaled and merged using Aimless^78^ as implemented in the CCP4 suite of crystallographic software^79^. Galectin-4N protein sequence (aa. 1–155) was used to build a molecular replacement model via the SWISS-MODEL server^80^ with mouse galectin-4N structure (PDB ID: 3I8T^55^) as template. The structures were solved by molecular replacement using the homology model with Phaser^81^ and were refined using Refmac5^82^. Visualization of electron density and model-building was performed using Coot^83^,^84^ and the models were validated and analysed by Molprobity^85^.

### Measurement of galectin-4N affinity toward oligosaccharides

A competitive fluorescence anisotropy (FA) inhibition assay^86^ was used to measure the affinity of galectin-4N towards a panel of saccharide ligands. A fixed concentration of a fluorescein tagged thio-di-galactoside derivative (0.1 μM) was mixed with 0.8 μM of galectin-4N and a dilution series of inhibitor ranging from about 20–2000 μM, and anisotropy was measured at room temperature using a PHERAstar plate reader (excitation 485 nm/emission 520 nm) with software PHERAstar Mars version 2.10 R3 (BMG, Offenburg, Germany). Kd-values were calculated as described^86^ and applied to galectin-4N and galectin-4C^50^,^87^. Each value is based on 6–8 measurements.

## Additional Information

**Accession codes**: Protein Data Bank: Atomic coordinates and structure factors have been deposited with accession codes for galectin-4N CRD with bound glycerol (5DUU), lactose (5DUV), 3′SuL (5DUW) and 2′FL (5DUX).

**How to cite this article**: Khuchtumur, B.-E. *et al.* Structural characterisation of human galectin-4 *N*-terminal carbohydrate recognition domain in complex with glycerol, lactose, 3′-sulfo-lactose, and 2′-fucosyllactose. *Sci. Rep.* **6**, 20289; doi: 10.1038/srep20289 (2016).

## Figures and Tables

**Table 1:** X-ray crystallographic data collection and refinement statistics of galectin-4N-ligand complexes.

	Galectin-4N CRD in complex with glycerol	Galectin-4N CRD in complex with lactose	Galectin-4N CRD in complex with 3′SuL	Galectin-4N CRD in complex with 2′FL
Crystal system, Space group	Monoclinic, *P*2_1_	Monoclinic, *P*2_1_	Monoclinic, *P*2_1_	Monoclinic, *P*2_1_
Unit cell parameters (Å)	*a* = 63.24,	*a* = 62.92,	*a* = 63.18,	*a* = 63.15,
	*b* = 64.53,	*b* = 64.75,	*b* = 64.05,	*b* = 64.47,
	*c* = 64.68,	*c* = 64.47,	*c* = 64.65,	*c* = 64.65,
	β = 90.88	β = 91.26	β = 91.03	β = 90.79
Resolution (Å)	45.68–2.00 (2.05–2.00)	37.24–1.90 (1.94–1.90)	64.64–1.70 (1.73–1.70)	45.65–1.85 (1.89–1.85)
Total number of observations	159500 (11018)	161697 (6854)	224805 (10361)	177950 (8997)
Total number of unique observations	35098 (2534)	40168 (2128)	56576 (2931)	44028 (2626)
Redundancy	4.5 (4.3)	4.0 (3.2)	4.0 (3.5)	4.0 (3.4)
Completeness (%)	99.5 (97.4)	98.1 (82.1)	99.5 (98.5)	99.1 (96.2)
I/σ (I)	15.8(2.8)	12.2(4.1)	13.7 (2.0)	22.9 (5.9)
R_merge_ (%)	6.4 (24.3)	6.7 (22.4)	6.3 (31.5)	3.6 (10.8)
*Refinement*				
Resolution (Å)	64.68–2.00 (2.05–2.00)	64.45–1.90 (1.95–1.90)	64.64–1.70 (1.74–1.70)	64.65–1.85 (1.90–1.85)
Total number of unique observations	33349 (2394)	38075 (2322)	53758 (2795)	41855 (2156)
*R*_Cryst_ (%)	18.4 (20.1)	15.5 (19.9)	15.0 (22.2)	16.6 (20.1)
*R*_free_ (%)	22.2 (23.9)	20.7 (24.7)	18.1 (23.7)	20.5 (24.2)
*No. atoms*				
Protein	4450	4478	4457	4414
Ligand	24	92	108	66
Water molecules	306	380	559	439
*Average B factors (Å*^*2*^)				
Protein	18.3	16.9	15.9	17.4
Ligand	20.4	16.6	15.4	28.2
Water molecules	24.2	24.5	26.7	26.5
*RMS deviations*				
Bond lengths (Å)	0.009	0.006	0.006	0.006
Bond angles (^o^)	1.34	1.06	1.21	1.11
*Ramachandran Plot Statistics*				
Favoured (%)	99.5	99.1	99.3	98.5
Allowed (%)	0.5	0.9	0.7	1.5
PDB ID	5DUU	5DUV	5DUW	5DUX

Values in parentheses are for the highest resolution shell. The Rfree value test set size is 5%.
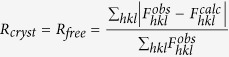

**Table 2:** Binding affinity of galectin-4N towards various saccharides.

Saccharides	Trivial Name	K_d_ (μM)	SEM
**Galβ1-4Glc**	Lactose	1300	90
SO_3_^−^-3**Galβ1-4Glc**	Lactose-3′-sulfate (3′SuL)	510	30
Fucα1–2**Galβ1-4Glc**	2′-fucosyllactose (2′FL)	380	9
GalNAc α1-3(Fucα1-2)**Galβ1-4Glc**	A-tetrasaccharide	164	5

The saccharide in bold most likely resides in sub-site C-D of the galectin CRD, as based on structural considerations and a value of A_max_ equal or higher than that for lactose itself, as discussed in detail elsewhere^66^,^88^.
